# Supplementary figures and images for: Mechanisms for dysregulation of excitatory-inhibitory balance underlying allodynia in dorsal horn neural subcircuits
Source: PLoS Comput Biol. 2025 Jan 14;21(1):e1012234. doi: 10.1371/journal.pcbi.1012234 (PMC11771949; doi:10.1371/journal.pcbi.1012234)

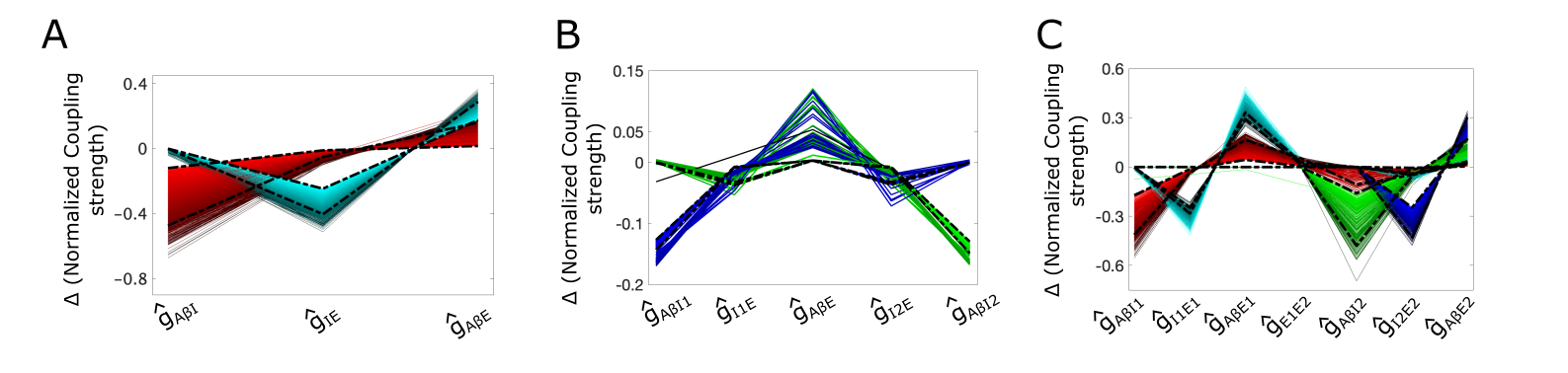

Supplement: S1 Fig — Shortest paths (solid lines) from each APS point to points on the allodynia surfaces whose distances correspond to a local minimum of distance no more than 1.5 times the global minimum distance. Global minima are included in these plots. Panels (A), (B), and (C) correspond respectively to the simple, static, and dynamic circuits. Clustering based on these local minima paths yields clusters analogous to clusters based on the global shortest paths. However, the range of displacements based on local minima paths is larger than the range of displacements based on global shortest paths (indicated by the region between the dashed-dotted lines), particularly for the static subcircuit. Notably, one local minima path for the static subcircuit and one for the dynamic subcircuit (solid black lines) do not fall into any of the identified clusters. (TIFF) [file pcbi.1012234.s001.tiff]

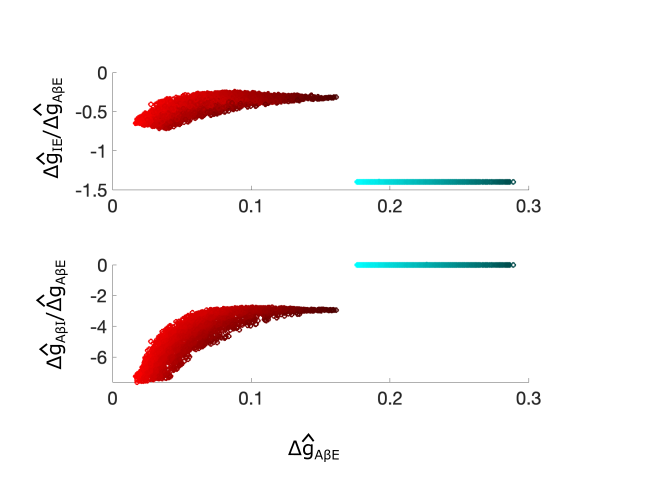

Supplement: S2 Fig — Ratios of components of the shortest path vectors in the g^IE-direction (top) and g^AβI-direction (bottom) relative to the g^AβE-direction versus the component in the g^AβE-direction across clusters in the APS for the simple subcircuit. We see that for the cyan cluster (Cluster 2) but not for the red cluster (Cluster 1), these ratios are fixed, indicating that the shortest paths to the allodynia surface are always in the same direction. (TIFF) [file pcbi.1012234.s002.tiff]
