# Supplementary material for: Mechanisms for dysregulation of excitatory-inhibitory balance underlying allodynia in dorsal horn neural subcircuits
Source: PLoS Comput Biol. 2025 Jan 14;21(1):e1012234. doi: 10.1371/journal.pcbi.1012234 (PMC11771949; doi:10.1371/journal.pcbi.1012234)
Supplement: S1 Appendix — (PDF) [file pcbi.1012234.s003.pdf]

Mechanisms for dysregulation of excitatory-inhibitory balance underlying allodynia in dorsal horn neural subcircuits

Alexander G. Ginsberg[1], Scott F. Lempka[2, 3, 4], Bo Duan[5], Victoria Booth[1,3], and Jennifer Crodelle[6],

1 Department of Mathematics, University of Michigan, Ann Arbor, Michigan, United States of America
2 Department of Biomedical Engineering, University of Michigan, Ann Arbor, Michigan, United States of America
3 Department of Anesthesiology, University of Michigan, Ann Arbor, Michigan, United States of America
4 Biointerfaces Institute, University of Michigan, Ann Arbor, Michigan, United States of America
5 Department of Molecular, Cellular and Developmental Biology, University of Michigan, Ann Arbor, Michigan, United States of America
6 Department of Mathematics and Statistics, Middlebury College, Middlebury, Vermont, United States of America.

S1 Appendix. Supplemental information for simplifying the inequalities that define the APS

Section A. Using Lambert-W functions to optimize $\frac{a\tanh(x-b)+c}{x}$.

To solve the optimization problems contained in the condition that specify reasonable behaviors for circuits such as the simple, dynamic and static subcircuits, it is necessary to find the critical points x_0, x_{-1} of

$$\frac{a\tanh(x-b)+c}{x}.$$

Such critical points are given by the 0 and -1 branch W_0 and W_{-1} of the Lambert-W function (Eq 6):

$$x_{0,-1} = \frac{1}{2}\left(c/a - W_{0,-1}\left(-e^{1-2b}\right)\right),$$

as long as $c = a$.

To show this, we solve

$$\begin{aligned}0 &= \frac{d}{dx}\left[\frac{a\tanh(x-b)+c}{x}\right]\\ &= \frac{a\ \mathrm{sech}^2(x-b)x - (a\tanh(x-b)+c)}{x^2}.\end{aligned}$$

Multiplying through by x^2, we thus need that

$$0 = a\ \mathrm{sech}^2(x-b)x - a\tanh(x-b) - c.$$

and thus that

$$0 = \ \mathrm{sech}^2(x-b)x - \tanh(x-b) - c/a,$$

which denoting c/a by d becomes

$$\begin{aligned}0 &= \ \mathrm{sech}^2(x-b)x - \tanh(x-b) - d\\ &= \ \mathrm{sech}^2(x-b)(x-b) + b\ \mathrm{sech}^2(x-b) - \tanh(x-b) - d\end{aligned}$$

which denoting $u = x - b$ becomes

$$0 = u\ \mathrm{sech}^2(u) + b\ \mathrm{sech}^2(u) - \tanh(u) - d.$$

Multiplying through by $\cosh^2(x-b)$, we then have that, denoting "if and only if" by "$\Leftrightarrow$",

$$\begin{aligned}0 &= u + b - \sinh(u)\cosh(u) - d\cosh^2(u)\\ &= u + b - \sinh(2u)/2 - d(1+\cosh(2u))/2 \Leftrightarrow\\ 0 &= 2u + 2b - d(1+\cosh(2u)) - \sinh(2u)\\ &= 2u + (2b-d) - d\cosh(2u) - \sinh(2u)\\ &= 2u + (2b-d) - d\left(e^{2u}+e^{-2u}\right)/2 - \left(e^{2u}-e^{-2u}\right)/2 \Leftrightarrow\\ 0 &= 4u + (4b-2d) - d\left(e^{2u}+e^{-2u}\right) - \left(e^{2u}-e^{-2u}\right).\end{aligned}$$

Multiplying through by e^{-2u}, we obtain

$$\begin{aligned}
0 &= 4ue^{2u} + (4b-2d)e^{2u} - d\left(e^{4u}+1\right) - \left(e^{4u}-1\right) \\
&= 4ue^{2u} + (4b-2d)e^{2u} + e^{4u}(-d-1) + (1-d) \\
&= (4u+4b-2d)e^{2u} - e^{4u}(1+d) + (1-d) \Leftrightarrow \\
0 &= \left(e^{d-2b}(2u+2b-d)\right)e^{2u+2b-d} - \left(\frac{1+d}{2}e^{2d-4b}\right)e^{4u+4b-2d} + (1-d)/2 \\
&= \frac{1-d}{2} + \left(e^{d-2b}\right)ve^{v} - \left(\frac{1+d}{2}e^{2d-4b}\right)e^{2v} \Leftrightarrow \\
0 &= \left(\frac{1-d}{2}e^{2b-d}\right) + ve^{v} - \left(\frac{1+d}{2}e^{d-2b}\right)e^{2v}.
\end{aligned}$$

If $d = 1$, the preceding equation simplifies to the following:

$$\begin{aligned}
0 &= ve^{v} - e^{1-2b}e^{2v} \Leftrightarrow \\
0 &= ve^{2b-1} - e^{v} \Leftrightarrow \\
0 &= e^{2b-1} - \frac{1}{v}e^{v} \Leftrightarrow \\
e^{2b-1} &= \frac{1}{v}e^{v} \Leftrightarrow \\
e^{1-2b} &= ve^{-v} \Leftrightarrow \\
-e^{1-2b} &= -ve^{-v}.
\end{aligned}$$

Then, as long as

$$\begin{aligned}
-e^{1-2b} &\geq -e^{-1} \Leftrightarrow \\
e^{1-2b} &\leq e^{-1} \Leftrightarrow \\
1-2b &\leq -1 \Leftrightarrow \\
2 &\leq 2b \Leftrightarrow \\
1 &\leq b,
\end{aligned}$$

the preceding equation, $-e^{1-2b} = -ve^{-v}$, has a solution given by the principal (W_0) and -1 (W_{-1}) branches of the Lambert-w function:

$$\begin{aligned}
W_{0,-1}\left(-e^{1-2b}\right) &= -v \Leftrightarrow \\
v &= -W_{0,-1}\left(-e^{1-2b}\right) \Leftrightarrow \\
2u+2b-d &= -W_{0,-1}\left(-e^{1-2b}\right) \Leftrightarrow \\
2u &= d-2b-W_{0,-1}\left(-e^{1-2b}\right) \Leftrightarrow \\
x-b &= \frac{1}{2}\left(d-2b-W_{0,-1}\left(-e^{1-2b}\right)\right) \Leftrightarrow \\
x &= b + \frac{1}{2}\left(d-2b-W_{0,-1}\left(-e^{1-2b}\right)\right) \\
&= b + \frac{1}{2}\left(c/a-2b-W_{0,-1}\left(-e^{1-2b}\right)\right).
\end{aligned}$$

Otherwise, the equation $-e^{1-2b} = -ve^{-v}$ has no solution, and there are no local extrema of the function being minimized. However if $d \neq 1$, i.e. if $c \neq a$, then there does not appear to be a general solution to the equation in terms of known functions. The solution to Eq 5 thus occurs at

$$x_{0,-1} = \frac{1}{2}\left(c/a - W_{0,-1}\left(-e^{1-2b}\right)\right),$$

so long as x_0 or $x_{-1} \in [f_{A\beta,min}, f_{A\beta,max}]$ or at $x = f_{A\beta,min}$ or $x = f_{A\beta,max}$, and $c = a$.

If instead $c \neq a$, we claim that $\frac{a\tanh(x-b)+c}{x}$ may have at most three critical points.

Indeed, we know from the preceding proof that the extrema occur when

$$0 = q(u) := (1-d) + (4u + 4b - 2d)e^{2u} - (1+d)e^{4u}.$$

However, it turns out that $q(u)$ can be zero at most three times. To see why, we will show that $q'(u)$ has at most two extremum on $(-\infty, \infty)$. Indeed,

$$\begin{aligned} q'(u) &= (8u + 8b - 4d + 4)e^{2u} - 4(1+d)e^{4u} \\ &= 4e^{2u}(2u + 2b - d + 1 - (1+d)e^{4u}), \end{aligned}$$

which is zero only when

$$\begin{aligned} (1+d)e^{4u} &= 2u + 2b - d + 1 \Leftrightarrow \\ e^{4u} &= \frac{2u + 2b - d + 1}{1+d}. \end{aligned}$$

Now, make the substitution

$$r := 2u + 2b - d + 1.$$

Then,

$$\begin{aligned} e^{2r-4b+2d-2} &= \frac{r}{1+d} \Leftrightarrow \\ (1+d)e^{-4b+2d-2} &= re^{-2r} \Leftrightarrow \\ -2(1+d)e^{-4b+2d-2} &= -2re^{-2r}. \end{aligned}$$

However, we can write the solution to the preceding expression using the principal W_0 and -1 W_{-1} branches of the Lambert-W function

$$\begin{aligned} -2r &= W_{0,-1}\left(-2(1+d)e^{-4b+2d-2}\right) \Leftrightarrow \\ r &= -\frac{1}{2}W_{0,-1}\left(-2(1+d)e^{-4b+2d-2}\right). \end{aligned}$$

Rewriting r back in terms of u, we have

$$\begin{aligned} 2u + 2b - d + 1 &= -\frac{1}{2}W_{0,-1}\left(-2(1+d)e^{-4b+2d-2}\right) \Leftrightarrow \\ u_{0,-1} &= \frac{1}{2}(-2b + d - 1) - \frac{1}{4}W_{0,-1}\left(-2(1+d)e^{-4b+2d-2}\right). \end{aligned}$$

In each of $(-\infty, \min\{u_0, u_{-1}\}]$, $(\max\{u_0, u_{-1}\}, \max\{u_0, u_{-1}\}]$, $(\max\{u_0, u_{-1}\}, \infty)$, q is monotonic, and thus can have at most one zero. Thus, q has at most three zeros. Moreover to search for critical points, we only need to find zeros in those three intervals, which is a fairly easy task because q is monotonic in the intervals. Once we find the zeros of $q(u)$ in those intervals, we substitute $u = x - b$, and compute $\frac{a\tanh(x-b)+c}{x}$ at each of the zeros of q and at the smallest and largest possible values of x. The largest and smallest of those values of $\frac{a\tanh(x-b)+c}{x}$ are thus the maximum and minimum values of $\frac{a\tanh(x-b)+c}{x}$.

Section B. Simplifying $V_{E,min} \leq g_{A\beta E}f_{A\beta} - g_{IE}f_I + V_{E,rest}$ by re-writing it as an optimization problem for $\frac{x+A}{1+\tanh(x+B)}$

This simplification involves rewriting the inequalities of the form of e.g.

$$V_{E,min} \leq g_{A\beta E}f_{A\beta} - g_{IE}f_I + V_{E,rest},$$

as in the middle three inequalities from Eq 4/Table 1, (also expressed in lines 3-4 of Table 6 for the simple subcircuit), and as in the $I1$ and $I2$ ablation conditions from Table 7 for the static subcircuit. In particular, the preceding equation is rewritten so it gives bounds on g_{IE} in terms of f_I and $g_{A\beta I}$:

$$\max_{f_{A\beta}} \frac{g_{A\beta E}f_{A\beta} + V_{E,rest} - V_{E,min}}{f_I} \leq g_{IE}. \qquad (1)$$

Eq (1) can be further rewritten as:

$$\begin{aligned}&\frac{g_{A\beta E} f_{A\beta} + V_{E,rest} - V_{E,min}}{0.5\max_I\left(1+\tanh((g_{A\beta I} f_{A\beta} + V_{I,rest} - \beta_I)/\alpha_I)\right)}\\&= \frac{g_{A\beta E}\alpha_I}{0.5\max_I g_{A\beta I}} \frac{g_{A\beta I} f_{A\beta}/\alpha_I + (V_{E,rest} - V_{E,min})/(g_{A\beta E}\alpha_I)}{1+\tanh((g_{A\beta I} f_{A\beta} + V_{I,rest} - \beta_I)/\alpha_I)},\end{aligned}$$

whose local extrema are shared with the following:

$$\frac{g_{A\beta I} f_{A\beta}/\alpha_I + (V_{E,rest} - V_{E,min})/(g_{A\beta E}\alpha_I)}{1+\tanh((g_{A\beta I} f_{A\beta} + V_{I,rest} - \beta_I)/\alpha_I)} = \frac{x+A}{1+\tanh(x+B)}.$$

Consequently, the solutions to the optimization problems of the form presented in (Eq (1)) occur either at $f_{A\beta,min}$, $f_{A\beta,max}$, or at the $f_{A\beta}$ corresponding to one of the critical points which solves

$$0 = \frac{d}{dx}\left[\frac{x+A}{1+\tanh(x+B)}\right], \tag{2}$$

where

$$\begin{aligned}x &= g_{A\beta I} f_{A\beta}/\alpha_I,\\ A &= (V_{E,rest} - V_{E,min})/(g_{A\beta E}\alpha_I),\\ B &= (V_{I,rest} - \beta_I)/\alpha_I.\end{aligned}$$

We show in Section C that the solution to Eq (2) is expressed in terms of the principal branch W_0 of the Lambert-W function:

$$x_0 = -\frac{1}{2}\left(1 + 2A + W_0\left(e^{2B-2A-1}\right)\right). \tag{3}$$

Writing x in terms of $f_{A\beta}$, the maximum referred to in the left-hand-side of Eq 1 occurs either when $f_{A\beta}$ is $f_{A\beta,min}$, $f_{A\beta,max}$, or

$$f_{A\beta,0} = -\frac{\alpha_I}{2g_{A\beta I}}\left(1 + 2A + W_0\left(e^{2B-2A-1}\right)\right),$$

so long as $f_{A\beta,0} \in [f_{A\beta,min}, f_{A\beta,max}]$. We use the solution (Eq (3)) to Eq (2) to make sampling from the static subcircuit more computationally efficient.

Section C. Using Lambert-W functions to optimize $\frac{x+A}{1+\tanh(x+B)}$.

To solve the optimization problems contained in conditions that subcircuit parameters need to satisfy to produce the desired behaviors, it is helpful to find the critical point x_0 of

$$\frac{x+A}{1+\tanh(x+B)}.$$

Such a critical point is given by the principal branch W_0 of the Lambert-W function (Eq (3)):

$$x_0 = -\frac{1}{2}\left(1 + 2A + W_0\left(e^{2B-2A-1}\right)\right).$$

Indeed, to solve Eq (2), we need to solve

$$\begin{aligned}0 &= \frac{d}{dx}\left[\frac{x+A}{1+\tanh(x+B)}\right]\\ &= \frac{1+\tanh(x+B) + \operatorname{sech}^2(x+B)(x+A)}{(1+\tanh(x+B))},\end{aligned}$$

which, multiplying through by the denominator and taking x and B to be positive so $\tanh(x+B) \neq -1$, occurs if and only if ($\Leftrightarrow$)

$$0 = 1 + \tanh(x+B) + (x+A)\operatorname{sech}^2(x+B).$$

Multiplying through by $\cosh^2(x+B)$, we can simplify the preceding expression so it can be solved using the Lambert-W function:

$$\begin{aligned}
0 &= \cosh^2(x+B) + \sinh(x+B)\cosh(x+B) + (x+A) \\
&= \frac{1}{2}\left(\cosh(2x+2B)+1\right) + \frac{1}{2}\sinh(2x+2B) + (x+A) \\
&= x + A + \frac{1}{2} + \frac{1}{2}\left(\cosh(2x+2B) + \sinh(2x+2B)\right) \\
&= x + A + \frac{1}{2} + \frac{1}{4}\left(2e^{2x+2B}\right) \\
&= x + A + \frac{1}{2} + \frac{1}{2}e^{2x+2B} \Leftrightarrow \\
0 &= 2x + 2A + 1 + e^{2x+2B} \Leftrightarrow \\
-1-2x-2A &= e^{2x+2B} \Leftrightarrow \\
1 &= \frac{1}{-1-2x-2A}e^{2x+2B} \Leftrightarrow \\
1 &= (-1-2x-2A)e^{-2x-2B} \\
&= (-1-2x-2A)e^{-2x-2A-1}e^{-2B+2A+1} \Leftrightarrow \\
e^{2B-2A-1} &= (-2x-2A-1)e^{-2x-2A-1}.
\end{aligned}$$

Noting that the left-hand side of the preceding equation is always positive, it follows that the preceding equation has its only real-valued solution given by the principal branch W_0 of the Lambert-W function

$$\begin{aligned}
-2x-2A-1 &= W_0\left(e^{2B-2A-1}\right) \Leftrightarrow \\
-2x &= 1 + 2A + W_0\left(e^{2B-2A-1}\right).
\end{aligned}$$

The solution to Eq (2) is thus

$$x_0 = -\frac{1}{2}\left(1 + 2A + W_0\left(e^{2B-2A-1}\right)\right).$$
