# Supplementary material for: Mechanisms for dysregulation of excitatory-inhibitory balance underlying allodynia in dorsal horn neural subcircuits
Source: PLoS Comput Biol. 2025 Jan 14;21(1):e1012234. doi: 10.1371/journal.pcbi.1012234 (PMC11771949; doi:10.1371/journal.pcbi.1012234)
Supplement: S2 Appendix — (PDF) [file pcbi.1012234.s004.pdf]

Mechanisms for dysregulation of excitatory-inhibitory balance underlying allodynia in dorsal horn neural subcircuits

Alexander G. Ginsberg[1], Scott F. Lempka[2, 3, 4], Bo Duan[5], Victoria Booth[1,3], and Jennifer Crodelle[6],

1 Department of Mathematics, University of Michigan, Ann Arbor, Michigan, United States of America
2 Department of Biomedical Engineering, University of Michigan, Ann Arbor, Michigan, United States of America
3 Department of Anesthesiology, University of Michigan, Ann Arbor, Michigan, United States of America
4 Biointerfaces Institute, University of Michigan, Ann Arbor, Michigan, United States of America
5 Department of Molecular, Cellular and Developmental Biology, University of Michigan, Ann Arbor, Michigan, United States of America
6 Department of Mathematics and Statistics, Middlebury College, Middlebury, Vermont, United States of America.

S2 Appendix. Supplemental information for the volume-independent sampling algorithm

Section A. Implementation of the volume-independent sampling algorithm.

Recall that we are attempting to sample from a space (the APS) that can be described by a hierarchical set of inequalities, i.e:

- $a_1 \leq x_1 \leq b_1$
- $L_2(x_1) \leq x_2 \leq U_2(x_1)$ for $x_2 \in [a_2, b_2]$
- $L_3(x_1, x_2) \leq x_3 \leq U_3(x_1, x_2)$ for $x_3 \in [a_3, b_3]$

$\vdots$

- $L_n(x_1, x_2, \ldots, x_{n-1}) \leq x_n \leq U_n(x_1, x_2, \ldots, x_{n-1})$ for $x_n \in [a_n, b_n]$

for some labeling $x_1, x_2, \ldots x_n$ of the populations in the circuit, some real numbers $a_1 \leq b_1, \ldots a_n \leq b_n$, and some functionals $L_1 \leq U_1, \ldots, L_n \leq U_n$.

To implement the algorithm for uniformly sampling from this region, described in Section 4.6, we uniformly select a hyperrectangle from a set of hyperrectangles that covers the APS via steps (1) - (3) below. Specifically, to randomly select the hyperrectangle, in steps (1) - (3) we randomly select the subintervals whose Cartesian product defines the hyperrectangle. As a result, we avoid the need to precompute the hyperrectangle-cover of the APS. In step (4), we randomly select a point from the hypercube. In steps (5) - (6), we randomly keep the point with probability proportional to the hyperrectangles volume, and then discard the point if it fails to fall in the APS. We describe the details below:

1. Partition $[a_1, b_1]$ into m_1 equal-sized subintervals
   - Uniformly at random select a subinterval.
   - Deterministically select a value of x_1 in that subinterval, e.g. by taking x_1 to be the left endpoint.
2. Given the value of x_1, compute $L_2(x_1)$ and $U_2(x_1)$.
   - If $L_2(x_1) \geq U_2(x_1)$, start over.
   - Otherwise, partition $[L_2(x_1), U_2(x_1)]$ into m_2 equal-sized subintervals.
     – Append an interval of size $(U_2(x_1) - L_2(x_1))/m_2$ to either side of the partition.
   - Uniformly at random select a subinterval from the partition.
   - Deterministically select a value of x_2 in that sub-interval.
3. Repeat the preceding step for $x_3, \ldots, x_n$. We will have now obtained n subintervals whose cartesian product forms a hypercube
4. Uniformly at random select a point in the hypercube

5. Randomly keep the point with probability proportional to the volume of the hypercube. To do so,
   - Compute the maximal possible volume of the hypercube.
     - Because the whole normalized parameter space lies in the unit hypercube $[0,1]\times ... \times [0,1]$ in $\mathbb{R}^n$, and because the selected hypercube has maximal length in dimension j given by $[U_j(x_1, x_2, \dots, x_{j-1}) - L_j(x_1, x_2, \dots, x_{j-1})]/m_j \le 1/m_j$, the maximal possible volume of the hypercube is $\left(\Pi_{j=1}^n m_j\right)^{-1}$.
   - Randomly select a number in [0, 1]. If that number is smaller than the volume of the selected hypercube divided by the maximum possible volume of selected hyperrectangles, keep the point.
6. If the selected point is not in the allowable parameter space, discard it.

We show in Section C that the time-complexity of the implementation is much better than that of the naive algorithm, so long as for all $j = 1, .., n$, the probability that the upper bound U_j is larger than the lower bound L_j is close to 1. Indeed, we show that:

$$\text{Time complexity of selecting a point} = \begin{cases} O(n^2) & U_j \ge L_j \text{ always for } j = 1, 2, \dots, n \\ O\left(\frac{1}{\beta^{n-2}}\right) & U_j < L_j \text{ with probability } \beta > 0 \text{ for } j = 1, 2, \dots, n \text{ and } n \ge 3. \end{cases}$$

Section B. Proof that naive sampling is inefficient.

The naive sampling strategy has the potential to be computationally inefficient. Namely, checking if a point is in the APS requires computing $L_1, U_1, L_2, U_2, \dots L_n, U_n$, which, as discussed in Section C, takes $O(n^2)$ to do, where n is the number of dimensions. Further, after normalizing the APS so that it lies in the unit hypercube, as described in Section 4.6, the sampled point will be in the sample space with probability given by $volume(APS)$, and thus the number of iterations needed to find a point in the APS will be $1/volume(APS)$. Thus, the order of the naive algorithm is $O(n^2/volume(APS))$.

Heuristically, if the average width of the APS in each component direction is α for some $\alpha \in (0,1)$, then it is reasonable to expect that $volume(APS) \approx \alpha^n$, and the algorithm becomes $O(n^2/\alpha^n)$. Thus, the naive sampling algorithm is exponential in n.

Section C. Proof that the sampling algorithm is volume-independent and has time-complexity O(n^2) under ideal conditions.

In this section, we seek to show that the time complexity of running one iteration of the sampling algorithm we have constructed is

$$\text{Time complexity of selecting a point} = \begin{cases} O(n^2) & U_j \ge L_j \text{ always for } j = 1, 2, \dots, n \\ O\left(\frac{1}{\beta^{n-2}}\right) & U_j < L_j \text{ with probability } \beta > 0 \text{ for } j = 1, 2, \dots, n \text{ and } n \ge 3, \end{cases}$$

where n is the number of coupling strengths underlying the circuit.

In particular, below we show that selecting a hypercube in a cover of the allowable parameter space (APS) is $O(n^2)$, so long as the simple set of inequalities which we are using to define the APS is a "proper" description of the APS in the sense that the upper bounds on each coupling strength are indeed larger than the corresponding lower bounds. We then show that the time-complexity of sampling from the rectangle is $O(n)$ and then argue that the amount of times the algorithm requires us to toss out the point to ensure we are sampling uniformly from the APS is independent of n.

Indeed, step 1 can be accomplished by randomly selecting a number between 0 and 1, multiplying the number by m_1, taking the ceiling of the result, and using that as the index for a preallocated, linearly spaced partition of $[a_1, b_1]$. Hence step 1 is O(1).

Likewise, partitioning $[L_j(x_1, \dots, x_{j-1}), U_j(x_1, \dots, x_{j-1})]$ and selecting a value of x_j from the partition is O(1). However, the number of operations needed to compute L_j and U_j likely scales with the number of coordinates that L_j and U_j depend upon, which is $j-1$. Thus, the whole process of selecting x_j–computing L_j and U_j, partitioning $[L_j, U_j]$, randomly selecting a subinterval from the partition, and deterministically selecting an x_j from the subinterval–is $O(j)$.

Thus, if $U_j \ge L_j$ always, steps (1) - (3) collectively take:

$$\begin{aligned} \text{Order of steps (1) - (3)} &= O(1) + O(2) + \dots + O(n) \\ &= O(n^2). \end{aligned}$$

However if $U_n < L_n$ with some fixed probability β, then we need to recompute $x_1, \ldots x_{n-1}$, L_n, and U_n with probability $\beta \in (0,1)$. Thus, we would need to recompute U_n and L_n $O(\beta^{-1})$ times. However, we would need to recompute x_{n-1} an additional $O(\beta^{-1})$ times, as well. If $U_{n-1} < L_{n-1}$ with probability β as well, for each time we would need to recompute U_n and L_n, we would need to recompute U_{n-1} and L_{n-1} an additional $O(\beta^{-1})$ times, leading to a total of $O(\beta^{-2})$ times that we would need to recompute U_{n-1} and L_{n-1}. Thus, by induction, we would need to recompute U_{n-j} and L_{n-j} a total of $O(\beta^{-j})$ times. Since computing U_{n-j} and L_{n-j} once is $O(n-j)$, we would spend a total time $O((n-j)\beta^{-j})$ computing U_{n-j} and L_{n-j}. Thus, the total amount of time spent computing the bounds is

$$\text{Order of steps (1) - (3)} = O\left(\sum_{j=0}^{n-2}(n-j)\beta^{-j}\right).$$

However, we can simplify the preceding sum by replacing β^{-1} with some constant $\kappa \in (1,\infty)$ and splitting the resulting sum into two sums:

$$\sum_{j=0}^{n-2}(n-j)\kappa^j = n\sum_{j=0}^{n-2}\kappa^j - \sum_{j=0}^{n-2} j\kappa^j.$$

The former sum in the split is a geometric series and thus sums to:

$$n\sum_{j=0}^{n-2}\kappa^j = n\frac{\kappa^{n-1}-1}{\kappa-1}.$$

The latter sum in the split, on the other hand, is the derivative of a geometric series. Indeed:

$$\begin{aligned}\sum_{j=0}^{n-2} j\kappa^j &= \kappa\sum_{j=0}^{n-2} j\kappa^{j-1}\\ &= \kappa\sum_{j=0}^{n-2}\frac{d}{d\kappa}\left[\kappa^j\right]\\ &= \kappa\frac{d}{d\kappa}\left[\sum_{j=0}^{n-2}\kappa^j\right].\end{aligned}$$

Using the formula for the sum of a geometric series and differentiating we obtain:

$$\begin{aligned}\sum_{j=0}^{n-2} j\kappa^j &= \kappa\frac{d}{d\kappa}\left[\frac{\kappa^{n-1}-1}{\kappa-1}\right]\\ &= \frac{(n-2)\kappa^n-(n-1)\kappa^{n-1}+\kappa}{(\kappa-1)^2}.\end{aligned}$$

Adding the two sums together and simplifying, we obtain:

$$\begin{aligned}\sum_{j=0}^{n-2}(n-j)\kappa^j &= n\frac{\kappa^{n-1}-1}{\kappa-1} - \frac{(n-2)\kappa^n-(n-1)\kappa^{n-1}+\kappa}{(\kappa-1)^2}\\ &= n\frac{\kappa^n-\kappa^{n-1}-\kappa+1}{(\kappa-1)^2} - \frac{(n-2)\kappa^n-(n-1)\kappa^{n-1}+\kappa}{(\kappa-1)^2}\\ &= \frac{2\kappa^n + \text{ lower order terms}}{(\kappa-1)^2},\end{aligned}$$

which has complexity given by

$$\begin{aligned}\sum_{j=0}^{n-2}(n-j)\kappa^j &\sim O\left(\kappa^{n-2}\right)\\ &\sim O\left(\frac{1}{\beta^{n-2}}\right).\end{aligned}$$

However, in step (4), we need to uniformly at random select a point from the hyperrectangle, which requires $O(n)$ operations. Thus, the time complexity of randomly sampling one point from the allowable parameter space is:

$$\begin{aligned}
\text{Time complexity of selecting a point} &= O(n) + \text{Order of steps (1) - (3)} \\
&= \begin{cases} O(n+n^2) & U_j \geq L_j \text{ always for } j = 1,2,\ldots,n \\ O\left(n + \left(\frac{1}{\beta}\right)^{n-2}\right) & U_j < L_j \text{ with probability } \beta > 0 \text{ for } j = 1,2,\ldots,n \text{ and } n \geq 3 \end{cases} \\
&= \begin{cases} O(n^2) & U_j \geq L_j \text{ always for } j = 1,2,\ldots,n \\ O\left(\frac{1}{\beta^{n-2}}\right) & U_j < L_j \text{ with probability } \beta > 0 \text{ for } j = 1,2,\ldots,n \text{ and } n \geq 3. \end{cases}
\end{aligned}$$

In step (5), discarding a point from the hypercube with probability proportional to the volume of the hyperrectangle will require the algorithm to take additional iterations. In particular, it will add a number of iterations proportional to one divided by the ratio of the average rectangle volume and the max possible rectangle volume. However, we see no reason for such a ratio to depend on either $m_1, m_2, \ldots, m_n$ or on the number of dimensions as long as the maximum possible rectangle volume is chosen well. Indeed, if the APS was a hyperrectangle, then each selected hyperrectangle in the cover of the APS would have the same volume, and the ratio of the seelected hyperrectangle volume to the max hyperrectangle volume would be 1. Thus, each rectangle would be kept with probability 1, so the number of times we would need to repeat steps (1) - (4) added by discarding the point with probability proportional to the volume of the hyperrectangle would be O(1).

In step (6), discarding the point from the hyperrectangle if the point fails to fall in the APS may increase the number of iterations the algorithm takes. However, as long as the hyperrectangle cover of the APS is a good approximation of the APS, the probability that the point fails to fall in the APS is low. For instance, if the APS is itself a hyperrectangle, such a probability is 0. Thus, we expect step (6) to increase the number of iterations in a dimension-independent manner.

Section D. Proof that the sampling algorithm indeed produces a uniform sample.

Recall that we have constructed an algorithm for randomly and uniformly-in-space sampling that allowable parameter space (APS), in Section 4.6. To prove that the algorithm and implementation work, we first show that in steps (1) - (3) of the implementation, there are $m_1 \Pi_{j=1}^{n}(2 + m_{i_j})$ hyperrectangles that can be selected, and that these hyperrectangles are selected uniformly at random in each iteration of the sampling algorithm.

To start, the hyperrectangles that can be selected are the same across all iterations of the sample. These hyperrectangles in particular are specified as the Cartesian product of the n-subintervals selected in one iteration of our algorithm.

Indeed, recall that in the first step of an iteration of our algorithm, we partition $[a_1, b_1]$ into m_1 equal-sized subintervals and uniformly at random select a subinterval. However, uniformly at random selecting a subinterval amounts to uniformly at random selecting one endpoint c_1 of the form

$$c_1 = a_1 + d\frac{b_1 - a_1}{m_1} \text{ for any } d = 0\ldots, m_1 - 1$$

from the partition. The corresponding subinterval is

$$\left[c_1, c_1 + \frac{b_1 - a_1}{m_1}\right],$$

which forms the x_1 portion of the Cartesian product that specifies the hyperrectangle. In the next iteration of the algorithm, in selecting a subinterval from $[U_2(c_1) - L_2(c_1)]$, we uniformly at random select an endpoints of the form

$$c_2 = L_2(c_1) + d\frac{U_2(c_1) - L_2(c_1)}{m_2} \text{ for any } d = -1, 0\ldots, m_2,$$

for which the corresponding subinterval is

$$\left[c_2, c_2 + \frac{U_2(c_1) - L_2(c_1)}{m_2}\right],$$

which in turn forms the x_2 portion of the Cartesian product that specifies the hyperrectangle. Repeating this process, we select a hyperrectangle of the form:

$$\left[c_1, c_1 + \frac{b_1 - a_1}{m_1}\right] \times \left[c_2, c_2 + \frac{U_2(c_1) - L_2(c_1)}{m_2}\right] \times \cdots \times \left[c_n, c_n + \frac{U_n(c_1, \ldots c_{n-1}) - L_n(c_1, \ldots, c_{n-1})}{m_n}\right]$$

for any

$$\begin{aligned}
c_1 &= a_1 + d\frac{b_1 - a_1}{m_1} \text{ for any } d = 0\ldots, m_1 - 1 \\
c_2 &= L_2(c_1) + d\frac{U_2(c_1) - L_2(c_1)}{m_2} \text{ for any } d = -1, 0\ldots, m_2 \\
&\vdots \\
c_n &= L_n(c_1, \ldots, c_{n-1}) + d\frac{U_n(c_1, \ldots c_{n-1}) - L_n(c_1, \ldots, c_{n-1})}{m_n} \text{ for any } d = -1, 0\ldots, m_n.
\end{aligned}$$

Note that such hyperrectangles are disjoint. Moreover, from Bayes' law, any set $\{c_1, c_2, \ldots, c_n\}$ is selected with probability

$$\begin{aligned}
Pr\left(\{c_1, c_2, \ldots, c_n\}\right) &= Pr(c_1) \cdot Pr(c_2|c_1) \cdot \ldots \cdot Pr(c_n|c_1, \ldots, c_{n-1}) \\
&= \frac{1}{m_1} \cdot \frac{1}{m_2 + 2} \cdot \ldots \cdot \frac{1}{m_n + 2},
\end{aligned}$$

where the last step follows because each c_{i_j} is the left endpoint of a uniformly at random selected subinterval given values of $c_1, \ldots, c_{j-1}$. Thus, each set $\{c_1, c_2, \ldots, c_n\}$ is selected uniformly at random, and so each hyperrectangle is selected uniformly at random.

Having shown that each hyperrectangle is selected uniformly at random, we can now show that the algorithm selects points uniformly at random from the allowable parameter space. To see why, we can compute the probability distribution P of selected points by considering some point x in some hyperrectangle R_j. In particular, from the law of total probability:

$$\begin{aligned}
P(x) &= Pr(R_j \text{ is selected}) \cdot Pr(x \text{ is selected } |R_j \text{ is selected}) \cdot Pr(x \text{ is kept } |x \text{ is selected and } R_j \text{ is selected} \\
&= \left(\frac{1}{m_1} \cdot \frac{1}{m_2 + 2} \cdot \ldots \cdot \frac{1}{m_n + 2}\right) \cdot \frac{1}{\text{Volume}(R_j)} \cdot \frac{\text{Volume}(R_j)}{\text{Maximum Possible Rectangle Volume}} \\
&= \left(\frac{1}{m_1} \cdot \frac{1}{m_2 + 2} \cdot \ldots \cdot \frac{1}{m_n + 2}\right) \frac{1}{\text{Maximum Possible Rectangle Volume}},
\end{aligned}$$

which is constant. Therefore, steps (1) - (4) indeed select points randomly and uniformly in space within the set of hyperrectangles approximating the allowable parameter space.

Thus, if the union of hyperrectangles from which we sample points contains the APS, we can obtain a uniform-in-space sample of the APS simply by discarding any of the points contained in steps (1) - (4) that are not actually in the allowable parameter space. We show next that under the right conditions, it is in fact the case that such a union of hyperrectangles does contain the APS. Namely, if $U_2, U_3, \ldots, U_n$ and $L_2, L_3, \ldots, L_n$ change sufficiently slowly, and if $m_1, \ldots, m_n$ are sufficiently large, the set of hyperrectangles approximating the allowable parameter space contains the allowable parameter space.

Indeed, consider some point x in the APS, and denote its coordinates by $(x_1, \ldots, x_n)$. We now show that each x_1, x_2,... lie within the intervals we partitioned in steps (1) - (3) of the algorithm. Namely, because $x_1 \in [0, 1]$, there exists c_1 such that

$$x_1 \in \left[c_1, c_1 + \frac{b_1 - a_1}{m_1}\right].$$

We know that

$$L_2(x_1) \leq x_2 \leq U_2(x_1).$$

However, for x to fall in one of the desired rectangles, we want to show that x_2 falls between the upper and lower boundaries of the partition constructed in step (2) of the algorithm:

$$\left[L_2(c_1) - \frac{U_2(c_1) - L_2(c_1)}{m_2}, U_2(c_1) + \frac{U_2(c_1) - L_2(c_1)}{m_2}\right].$$

This is clearly the case if $x_1 = c_1$, since then we would have that

$$L_2(c_1) \leq x_2 \leq U_2(c_1).$$

Then assuming U_2 and L_2 change sufficiently slowly on $\left[c_1, c_1 + \frac{b_1 - a_1}{m_1}\right]$,

$$L_2(c_1) - \frac{U_2(c_1) - L_2(c_1)}{m_2} \leq L_2(x_1)$$
$$U_2(x_1) \leq U_2(c_1) + \frac{U_2(c_1) - L_2(c_1)}{m_2}$$

so

$$L_2(c_1) - \frac{U_2(c_1) - L_2(c_1)}{m_2} \leq x_2 \leq U_2(c_1) + \frac{U_2(c_1) - L_2(c_1)}{m_2}.$$

Likewise, if for each $j = 1, \ldots, n$ it's true that $U_{i_j}(c_1, \ldots, c_{j-1})$ and $L_{i_j}(c_1, \ldots, c_{j-1})$ vary sufficiently slowly, then

$$L_{i_j}(c_1, \ldots, c_{j-1}) - \frac{U_{i_j}(c_1, \ldots, c_{j-1}) - L_{i_j}(c_1, \ldots, c_{j-1})}{m_{i_j}} \leq x_2 \text{ and}$$
$$x_2 \leq U_j(c_{1, \ldots, c_{j-1}}) + \frac{U_{i_j}(c_1, \ldots, c_{j-1}) - L_{i_j}(c_1, \ldots, c_{j-1})}{m_{i_j}}.$$

Therefore, x lies in one of the desired hyperrectangles. Thus, if $U_1, \ldots, U_n$ and $L_1, \ldots, L_n$ change sufficiently slowly, and $m_1, \ldots, m_n$ are sufficiently large, then the hypercube approximation of the allowable parameter space is a good approximation of the actual allowable parameter space, and our random sampling algorithm produces a uniform sample of the allowable parameter space. □
