# Supplementary material for: Mechanisms for dysregulation of excitatory-inhibitory balance underlying allodynia in dorsal horn neural subcircuits
Source: PLoS Comput Biol. 2025 Jan 14;21(1):e1012234. doi: 10.1371/journal.pcbi.1012234 (PMC11771949; doi:10.1371/journal.pcbi.1012234)
Supplement: S3 Appendix — (PDF) [file pcbi.1012234.s005.pdf]

Mechanisms for dysregulation of excitatory-inhibitory balance underlying allodynia in dorsal horn neural subcircuits

Alexander G. Ginsberg[1], Scott F. Lempka[2, 3, 4], Bo Duan[5], Victoria Booth[1,3], and Jennifer Crodelle[6],

1 Department of Mathematics, University of Michigan, Ann Arbor, Michigan, United States of America
2 Department of Biomedical Engineering, University of Michigan, Ann Arbor, Michigan, United States of America
3 Department of Anesthesiology, University of Michigan, Ann Arbor, Michigan, United States of America
4 Biointerfaces Institute, University of Michigan, Ann Arbor, Michigan, United States of America
5 Department of Molecular, Cellular and Developmental Biology, University of Michigan, Ann Arbor, Michigan, United States of America
6 Department of Mathematics and Statistics, Middlebury College, Middlebury, Vermont, United States of America.

S3 Appendix. Supplemental information for the computationally efficient strategy for finding the distance betwen sampled points and the allodynia surface

Section A. Implementing the computationally efficient strategy for finding the distance between sampled points and the allodynia surface.

To implement our strategy for finding the distance between sampled points and the allodynia surface, as discussed in Section 4.8, we solve the original optimization problem (Eq 11) by finding a solution to the secondary optimization problem (Eq 12). In particular, recall that the optimization problem from Eq 12 is to find

$$\text{argmin}||\vec{gp} - \vec{g}||$$

over pairs $(\vec{g}, f_{in})$ subject to the conditions that

1. $g_{in,y} f_{in} = (V_{y,rest} - V_{y,thr}) + \sum_{j=1}^{n} g_{x_i y} f_{x_i}(\vec{g}, f_{in})$ for some $f_{in} \in [f_{in,min}, f_{in,max}]$, i.e. that a circuit with coupling strengths $\vec{g}$ and input signal f_{in} reaches the target state.

2. $g_{x_1 y}, \ldots, g_{x_n y}, g_{in,y} \geq 0$.

3. $f_{in} \in [f_{in,min}, f_{in,max}]$.

To solve the problem numerically, we use a multi-start stochastic global optimization scheme:

1. For efficiency, we restrict the search space by requiring that
   (a) The coupling strengths $\hat{g}_{x_1 y}, \ldots, \hat{g}_{x_n y}, \hat{g}_{in,y} \in [-1, 2]$.
   (b) Since we know that the desired $\hat{g}_{in,y} \geq gp_{in,y}$, we further restrict $\hat{g}_{in,y} \geq gp_{in,y}$.

2. We apply Matlab's stochastic gradient descent-based algorithm *fmincon* to the preceding optimization problem with the preceding extra restrictions.

3. We repeat the algorithm 15 times using start points selected uniformly at random from the preceding constraints, and take as the solution to the optimization problem the value of $\vec{g}$ yielding the smallest $||\vec{gp} - \vec{g}||$.

Section B. Proof that the primary and secondary optimization problems are equivalent.

Recall that the primary optimization problem (Eq 11) is to find the closest point $\vec{gs}$ on the allodynia surface S to the point $\vec{gp}$ in the allowable parameter space:

$$\vec{gs}_{nearest} = \text{argmin}_{\vec{gs} \in S} ||\vec{gp} - \vec{gs}||,$$

where we denote

$$\vec{gs}_{nearest} = (gs_{x_1 y}, \cdots, gs_{x_n y}, gs_{in,y}).$$

However, because of the difficulty in finding S, recall that we instead try to solve the secondary optimization problem (Eq 12):

$$(\vec{gs^*}_{nearest}, f_{in,nearest}) = \text{argmin}_{(\vec{gs},f_{in})\in S^*} ||\vec{gp} - \vec{gs}||,$$

where S^* is the following set of coupling strength-input signal pairs defined by removing the minimization from Eq 10:

$$S^* := \left\{ (\vec{g}, f_{in}) : g_{in,y} = \frac{(V_{y,rest} - V_{y,thr}) + \sum_{j=1}^{n} g_{x_i y} f_{x_i}(\vec{g}, f_{in})}{f_{in}} \text{ and } f_{in} \in [f_{in,min}, f_{in,max}] \right\},$$

and where going forward we will denote the solution $\vec{gs^*}_{nearest}$ as

$$\vec{gs^*}_{nearest} = (gs^*_{x_1 y}, \cdots, gs^*_{x_n y}, gs^*_{in,y}).$$

Thus, for the preceding strategy to work, we need

$$\vec{gs^*}_{nearest} = \vec{gs}_{nearest}.$$

Indeed, it can be proven that $\vec{gs^*}_{nearest} = \vec{gs}_{nearest}$ via the steps outlined below:

1. We show that the two optimization problems are "well-behaved" in that solutions to the primary and secondary optimization problems exist and lie in S and S^*, respectively.
2. We then provide a simpler way of showing that the solutions to the secondary optimization problem solve the original optimization problem. Namely, we show that $\vec{gs^*}_{nearest}$ solves the original optimization problem if and only if $\vec{gs^*}_{nearest} \in S$.
3. We establish that $\vec{gs^*}_{nearest} = \vec{gs}_{nearest}$ by showing that $\vec{gs^*}_{nearest} \in S$.

The details are as follows:

1. Lemma: $\vec{gs}_{nearest}$ exists and $\vec{gs}_{nearest} \in S$. Likewise, $\vec{gs^*}_{nearest}$ exists and $\vec{gs^*}_{nearest} \in S^\dagger$, the projection of S^* onto the space of coupling strengths:

$$S^\dagger := \left\{ \vec{g} : g_{in,y} = \frac{(V_{y,rest} - V_{y,thr}) + \sum_{j=1}^{n} g_{x_j y} f_{x_j}(\vec{g}, f_{in})}{f_{in}} \text{ for some } f_{in} \in [f_{in,min}, f_{in,max}] \right\}.$$

   Proof:

   (a) Since S is the graph of a continuous function on $\mathbb{R}$, it follows that S is a closed and connected n-dimensional manifold embedded in $\mathbb{R}^{n+1}$. Further, the minimization function $||gp - gs||$ is continuous and bounded away from infinity. Therfore, the map from $S \to \mathbb{R}$ given by $\vec{gs} \to ||\vec{gp} - \vec{gs}||$ is continuous on a bounded and connected set, and therefore maps to a bounded and connected set in $\mathbb{R}$ which has a maximum and attains the maximum for some $\vec{gs} \in S$. Thus $\vec{gs}_{nearest}$ exists, and $\vec{gs}_{nearest} \in S$.

   (b) Likewise, S^* is the graph of a continuous function on $\mathbb{R}^n \times [f_{in,min}, f_{in,max}]$ (where $g_{in,y}$ defines the height of the graph). Hence S^* is closed and connected, and so its projection $S^\dagger$ onto the space of coupling strengths is a closed $(n+1)$-dimensional manifold embedded in $\mathbb{R}^{n+1}$. Thus, following the argument given for $\vec{gs}_{nearest}$, $\vec{gs^*}_{nearest}$ exists, and $\vec{gs^*}_{nearest} \in S^\dagger$. □

2. Lemma: $\vec{gs^*}_{nearest}$ solves the original optimization problem Eq 11 if and only if ($\Leftrightarrow$) $\vec{gs^*}_{nearest} \in S$.

   (a) "$\Rightarrow$": If $\vec{gs^*}_{nearest}$ solves Eq 11, then, $\vec{gs^*}_{nearest} \in S$ by the preceding lemma.

   (b) "$\Leftarrow$": If $\vec{gs^*}_{nearest} \in S$, then by construction,

$$\min_{\vec{gs}\in S} ||\vec{g} - \vec{gs}|| \leq ||\vec{g} - \vec{gs^*}_{nearest}||.$$

   However, since $S \subset S^\dagger$ (as per the proof in (c), below),

$$\min_{(\vec{gs},f_{in})\in S^*} ||\vec{g} - \vec{gs}|| \leq \min_{\vec{gs}\in S} ||\vec{g} - \vec{gs}||.$$

   Thus,

$$\min_{\vec{gs}\in S} ||\vec{g} - \vec{gs}|| = ||\vec{g} - \vec{gs^*}_{nearest}||,$$

   and so $\vec{gs^*}_{nearest}$ solves the original minimization problem Eq 11. □

(c) Lemma for "⇐": $S \subset S^\dagger$. Proof:
$\vec{gs}$ satisfies

$$gs_{in,y} = \frac{(V_{y,rest} - V_{y,thr}) + \sum_{j=1}^{n} gs_{x_j y} f_{x_j}(\vec{g}, f_{in})}{f_{in}}$$

for some $f_{in} \in [f_{in,min}, f_{in,max}]$. Indeed, as long as $0 \notin [f_{in,min}, f_{in,max}]$, the function being maximized in Eq 10 is continuous and thus must attain its maximum at some $f_{in} \in [f_{in,min}, f_{in,max}]$. Hence, any point $gs \in S$ must also be in $S^\dagger$, and so $S \subset S^\dagger$. □

3. We claim that $\vec{gs^*}_{nearest}$ is the point $\vec{gc} \in S$ given by

$$\vec{gc} := \left(gs^*_{x_1 y}, \ldots, gs^*_{x_n y}, \min_{f_{in}} \left[\frac{(V_{y,rest} - V_{y,thr}) + \sum_{j=1}^{n} gs^*_{x_j y} f_{x_j}(\vec{gs^*}_{nearest}, f_{in})}{f_{in}} \right] \right).$$

If we can establish such a result, we will have shown that $\vec{gs^*}_{nearest} \in S$, and so $\vec{gs^*}_{nearest}$ solves the original optimization problem (Eq 11). To establish such a result, we will use that because $\vec{gc}$ and $\vec{gs^*}_{nearest}$ are vertically displaced from one another, $\vec{gc} = \vec{gs^*}_{nearest}$ if and only if

$$||\vec{gs^*}_{nearest} - \vec{gp}|| = ||\vec{gc} - \vec{gp}||.$$

To show this, we show (a) that $||\vec{gs^*}_{nearest} - \vec{gp}|| \leq ||\vec{gc} - \vec{gp}||$. We then show (b) that $||\vec{gs^*}_{nearest} - \vec{gp}|| \geq ||\vec{gc} - \vec{gp}||$ using the result shown in (c):

(a) Indeed, by the construction of $\vec{gs^*}_{nearest}$,

$$||\vec{gs^*}_{nearest} - \vec{gp}|| \leq ||\vec{gc} - \vec{gp}||.$$

(b) To show that $||\vec{gc} - \vec{gp}|| \geq ||\vec{gc} - \vec{gp}||$ we want to determine whether the following argument holds:

$$\begin{aligned}
||\vec{gc} - \vec{gp}|| &= \left\| \left(gs^*_{x_1 y}, \ldots, gs^*_{x_n y}, \min_{f_{in}} \left[\frac{(V_{y,rest} - V_{y,thr}) + \sum_{j=1}^{n} gs^*_{x_j y} f_{x_j}(\vec{gs^*}_{nearest}, f_{in})}{f_{in}} \right] \right) - \vec{gp} \right\| \\
&= \left\| \left(gs^*_{x_1 y} - gp_{x_1 y}, \ldots, gs^*_{x_n y} - gp_{x_n y}, \min_{f_{in}} \left[\frac{(V_{y,rest} - V_{y,thr}) + \sum_{j=1}^{n} gs^*_{x_j y} f_{x_j}(\vec{gs^*}_{nearest}, f_{in})}{f_{in}} \right] - gp_{in,y} \right) \right\| \\
&\leq || \left(gs^*_{x_1 y} - gs^*_{x_1 y}, \ldots, gs^*_{x_n y} - gp_{x_n y}, gs^*_{in,y} - gp_{in,y} \right) || \\
&= ||\vec{gs^*}_{nearest} - \vec{gp}||.
\end{aligned}$$

The issue is the third of the four equations listed above. It turns out that to show the third equation holds, it suffices to show that

$$gs^*_{in,y} - gp_{in,y} \geq \min_{f_{in}} \left[\frac{(V_{y,rest} - V_{y,thr}) + \sum_{j=1}^{n} gs^*_{x_j y} f_{x_j}(\vec{gs^*}_{nearest}, f_{in})}{f_{in}} \right] - gp_{in,y} \geq 0,$$

or equivalently, that

$$gs^*_{in,y} \geq \min_{f_{in}} \left[\frac{(V_{y,rest} - V_{y,thr}) + \sum_{j=1}^{n} gs^*_{x_j y} f_{x_j}(\vec{gs^*}_{nearest}, f_{in})}{f_{in}} \right] \geq gp_{in,y}.$$

To show that the former of such inequalities holds, note that because $\vec{gs^*}_{nearest} \in S^\dagger$, it follows that for some $f_{in} \in [f_{in,min}, f_{in,max}]$,

$$gs^*_{in,y} = \frac{(V_{y,rest} - V_{y,thr}) + \sum_{j=1}^{n} gs^*_{x_j y} f_{x_j}(\vec{gs^*}_{nearest}, f_{in})}{f_{in}}.$$

Hence,

$$gs^*_{in,y} \geq \min_{f_{in}} \left[\frac{(V_{y,rest} - V_{y,thr}) + \sum_{j=1}^{n} gs^*_{x_j y} f_{x_j}(\vec{gs^*}_{nearest}, f_{in})}{f_{in}} \right].$$

We show that the latter of such inequalities, i.e. that $\min_{f_{in}} \left[\frac{(V_{y,rest} - V_{y,thr}) + \sum_{j=1}^{n} gs^*_{x_j y} f_{x_j}(\vec{gs^*}_{nearest}, f_{in})}{f_{in}} \right] \geq gp_{in,y}$, holds in step (c), below, thereby completing the proof.

(c) Claim: it is always true that $\min_{f_{in}} \left[\frac{(V_{y,rest}-V_{y,thr})+\sum_{j=1}^{n} gs^*_{x_j y} f_{x_j}(\vec{gs^*}_{nearest}, f_{in})}{f_{in}}\right] \geq gp_{in,y}$. To prove this, we need only show that the point $(gs^*_{x_1 y}, \ldots, gs^*_{x_n y}, gp_{in,y})$, which is vertically displaced from $\vec{gc}$, lies on or beneath the allodynia surface. Proof:

Suppose not. Then, $(gs^*_{x_1 y}, \ldots, gs^*_{x_n y}, gp_{in,y})$ lies directly above the allodynia surface. However, by construction, $\vec{gp}$ lies below the allodynia surface. Thus, since the space above the allodynia surface and the space below the allodynia surface are not path connected (because the graph of any continuous function from $\mathbb{R}^n \to \mathbb{R}$ separates $\mathbb{R}^{n+1}$ into a space strictly above the graph and a space strictly below, which are not path connected with each other), it follows that the line segment joining $\vec{gp}$ and $(gs^*_{x_1 y}, \ldots, gs^*_{x_n y}, gp_{in,y})$ passes through the allodynia surface. In particular, there is a point on the line segment that is on the allodynia surface, with displacement from $\vec{gp}$ given by

$$\begin{aligned}\vec{e} &:= a\left[(gs^*_{x_1 y}, \ldots, gs^*_{x_n y}, gp_{in,y}) - \vec{gp}\right] \\ &= (gs^*_{x_1 y} - gp_{x_1 y}, \ldots, gs^*_{x_n y} - gp_{x_n y}, 0),\end{aligned}$$

for some $a \in (0, 1)$. Consequently,

$$\begin{aligned}||\vec{e}|| &= a||(gs^*_{x_1 y} - gp_{x_1 y}, \ldots, gs^*_{x_n y} - gp_{x_n y}, 0)|| \\ &< ||(gs^*_{x_1 y} - gp_{x_1 y}, \ldots, gs^*_{x_n y} - gp_{x_n y}, 0)|| \\ &\leq ||(gs^*_{x_1 y} - gp_{x_1 y}, \ldots, gs^*_{x_n y} - gp_{x_n y}, gs^*_{in,y} - gp_{in,y})|| \\ &= ||\vec{gs^*} - \vec{gp}||.\end{aligned}$$

This contradicts that $\vec{gs^*}$ is the closest point in $S^\dagger$ to $\vec{gp}$. Therefore, it must be the case that $(gs^*_{x_1 y}, \ldots, gs^*_{x_n y}, gp_{in,y})$ lies on or directly beneath the allodynia surface. □

Since $\vec{gc}$ lies on the surface and is vertically displaced from $(gs^*_{x_1 y}, \ldots, gs^*_{x_n y}, gp_{in,y})$, it follows that

$$\begin{aligned}gp_{in,y} &\leq \hat{gs^*}_{in,y} \text{ i.e. that} \\ gp_{in,y} &\leq \min_{f_{in}} \left[\frac{(V_{y,rest} - V_{y,thr}) + \sum_{j=1}^{n} gs^*_{x_j y} f_{x_j}(\vec{gs^*}, f_{in})}{f_{in}}\right]. \square\end{aligned}$$

Thus,

$$||\vec{gc} - \vec{gp}|| = ||\vec{gs^*} - \vec{gp}||.$$

However, $\vec{gc}$ and $\vec{gs^*}_{nearest}$ are vertically displaced from one other. Hence, $\vec{gc} = \vec{gs^*}_{nearest}$. Consequently, $\vec{gs^*}_{nearest} \in S$. So, it is indeed the case that $\vec{gs^*}_{nearest} = \vec{gs}_{nearest}$, and the two optimization problems are equivalent. □
